# The rational dimension of understanding

**DOI:** 10.1007/s11229-022-03839-z

**Published:** 2022-08-17

**Authors:** Miloud Belkoniene

**Affiliations:** Philosophy, University of Glasgow, Glasgow, UK

**Keywords:** Understanding, Justification, Rationality, Belief, Acceptance

## Abstract

It is natural to regard understanding as having a rational dimension, in the sense that understanding seems to require having justification for holding certain beliefs about the world. Some philosophers however argue that justification is not required to gain understanding of phenomena. In the present paper, my intention is to provide a critical examination of the arguments that have been offered against the view that understanding requires justification in order to show that, contrary to what they purport to establish, justification remains a plausible requirement on understanding.

## Introduction

It is natural to regard understanding as having a rational dimension, in the sense that understanding seems to require having justification for holding certain beliefs about the world. If one attributes to S an understanding of why *p*, one normally expects S to be able to offer an answer to the question “why is *p* the case?” that she has justification to endorse. In that, understanding appears to be similar to other valuable cognitive standings such as knowledge, although it may differ from knowledge in other respects.^1^ Kvanvig, for instance, claims that:

What is distinctive about understanding, once we have satisfied the truth requirement, is internal to cognition. It is the internal seeing or appreciating of explanatory and other coherence inducing relationships in a body of information that is crucial for understanding. (2003, p. 198).

The explanatory and coherence inducing relations Kvanvig is referring to here are naturally understood as support relations that obtain between the various elements of an account one possesses. For once one appreciates the explanatory relations between the elements of the account of why *p* one possesses, it is extremely plausible that one thereby appreciates how these elements support each other. Kelp (2015, p. 3810), who argues for a knowledge-based account of understanding, explicitly requires that the way one’s beliefs about the understood phenomenon are based reflects one’s knowledge of the support relations that obtain among the elements of the account of the understood phenomenon one possesses. Thus there is an intuition shared among certain philosophers who are interested in the nature of understanding that this particular cognitive standing has an important rational dimension.

Other philosophers such as Hills (2016) and Dellsén (2017, 2018, 2019) however argue that, contrary to what these intuitions suggest, justification is not required to gain understanding of phenomena. In the present paper, my intention is to provide a critical examination of the arguments that have been offered against the view that understanding requires justification in order to show that, contrary to what they purport to establish, understanding does involve a justification requirement. By focusing on explanatory understanding—that is, the kind of understanding typically promoted by explanations—I examine, in Sect. 2, arguments in favour of the claim that understanding can be based on defeated evidence. I argue that, contrary to what has been claimed in the recent literature, understanding is not compatible with epistemic defeat and that there are independent reasons to consider that for S to understand why *p* by means of an explanation H, S needs justification for believing H’s content. Section 3, for its part, discusses arguments related to deductive cogency as a requirement on belief. I argue that, contrary to what Dellsén (2018, 2019) claims, the fact that deductive cogency is a plausible requirement on the kind of propositional commitment involved in understanding does not provide compelling grounds for concluding that understanding does not requires justified belief.

## Arguments from epistemic defeat

### Evidence for the explanandum

Can understanding be based on defeated evidence? Consider the following case due to Dellsén:

*The Con Man*: Bernie is a retired automobile mechanic living in a very small town in rural America. One morning Bernie reads in the local newspaper that a convicted confidence man is coming to town. The story included a picture of the man and the following warning: ‘This man will try to scam you, so don’t believe a word he says.’ The next day, the con man is driving past Bernie’s house when his car suddenly breaks down. The con man rings on Bernie’s doorbell, and Bernie opens the door. Recognizing the con man’s face from the newspaper, Bernie decides to stay inside his house while conversing with the con man. The con man tells Bernie what appears to be wrong with the car and solicits Bernie’s assistance. Based only on the con man’s description of the car’s behaviour immediately before the breakdown (all of which is accurate), Bernie immediately diagnoses the problem as a broken timing belt (which is correct). Yet Bernie is not justified in believing this, since he should know better than to trust a convicted con man. (2017, pp. 243–244)

According to Dellsén, it is clear that Bernie understands what is wrong with the con man’s car although he lacks justification for believing what the con man told him. As a result, this case shows, in Dellsén’s view, that understanding does not require justification. Let me specify the understanding Bernie is supposed to have here as well as the propositions for which he lacks justification.

The Con Man case is designed to elicit the intuition that Bernie understands why the con man’s car broke down despite lacking justification for believing that the reason why the car exhibited behaviour *x* before breaking down is that a timing belt broke. Accordingly, this case looks like a case in which a subject S appears to understand why *p* by means of an account H although she is not justified in endorsing H’s content. But it is important to note that the reason why Bernie lacks justification for believing that the car exhibited behaviour *x* before breaking down because a timing belt broke is that he is not justified in believing that the car exhibited behaviour *x* before breaking down in the first place. More precisely, as the con man is known by Bernie to be untrustworthy, Bernie is neither justified in believing that the car exhibited behaviour *x* nor is he justified in believing that the car broke down. It follows that The Con Man case is really a case in which the subject who supposedly understands why *p* is not justified in believing that *p* is the case.

As outlined by Dellsén (2017, p. 244), there are two possible readings of the case when it comes to Bernie’s lack of justification for believing that the car exhibited behaviour *x* before breaking down. On the first reading which relies on a reductionist view of testimony, the con man’s testimony, because of what Bernie knows of the con man, does not provide Bernie with a reason to believe that the car exhibited behaviour *x* before breaking down. On this reading, the reason why Bernie lacks justification is therefore that he lacks a reason for believing that the car exhibited behaviour *x* before breaking down. On the second reading, the testimony provides Bernie with a defeasible reason for believing that the car exhibited behaviour *x* before breaking down but that reason is defeated by what Bernie read about the con man in the newspapers. That is, what Bernie read in the newspapers *undercuts* the reason provided by the con man’s testimony in favour of the claim that the car exhibited behaviour *x* before breaking down. Here I do not think that much hinges on preferring one reading over the other and, therefore, I will leave the question open as to whether Bernie simply lacks reasons for believing that the car exhibited behaviour *x* before breaking down or has reasons which are defeated by independent considerations. What matters is whether Bernie can understand why the car exhibited behaviour *x* before breaking down without being justified in believing that this is the case.

What follows, precisely, from the fact that Bernie lacks justification for believing that the car exhibited behaviour *x* before breaking down? One direct consequence of Bernie’s epistemic situation is that Bernie is not justified in believing that there is a correct explanation of the state of affairs reported by the con man. For if a subject is not justified in believing that *p* is the case, then she cannot be justified in believing that there is a correct explanation of *p*. There being a correct (and not merely possible) explanation of *p* entails that *p* is the case. Bernie, thus, lacks justification for believing that there is a correct explanation of why the car exhibited behaviour *x* before breaking down. Now, is this compatible with being in a position to understand why the car exhibited behaviour *x* before breaking down? Take the following situation:

*Brain Lesion*: Sam is sitting at his desk away from his kitchen. He suffers from a particularly strange type of brain lesion which makes him form beliefs about various states of affairs he has no reason to hold. Due to this brain lesion, Sam forms the belief that a glass fell on the kitchen floor and broke. He has absolutely no reason to hold this belief as he neither heard nor saw anything indicating that a glass fell on the kitchen floor and broke. Yet, his lesion-induced belief happens to be correct and Sam, who is quite knowledgeable when it comes to the kind of physical forces that are responsible for a glass to break in such conditions, endorses a correct account of why the glass that just fell on the kitchen floor broke.

In such a situation, most would be reluctant to attribute Sam with an understanding of why the glass that fell on the kitchen floor broke. This is because, although Sam is compelled by his medical condition to believe that a glass broke on his kitchen floor, he has absolutely no reason to believe that there is something to explain in the first place. The problem here is not Sam’s grasp of the explanatory link between the account he comes to endorse and the content of his lesion-induced belief, for Sam’s grasp of that link can be assumed to be ideal. What matters when it comes to attributing Sam with an understanding of why the glass broke is that he lacks justification for believing that what prompts him to endorse a particular account is in need of being explained.

Of course, in the Brain Lesion case, there is no question of epistemic defeat as Sam has no reason to believe that a glass fell on his kitchen floor and broke. But it is easy to modify the case in order to make it fit the second possible reading of Dellsén’s case. Consider the following version of the case similar to a situation discussed by Grimm (2006, p. 520):

*Hallucinatory Drug*: Sam has been drugged and has undergone various hallucinations since he drank the coffee which contained the drug. This has led him to the conclusion that one of his friends drugged his coffee and, after calling the friend at issue, he received confirmation that his coffee was drugged. Still under the influence of the drug, Sam hallucinates a glass falling on the floor of his kitchen and breaking. But this time, by sheer luck, a glass really fell on his kitchen floor and broke. Sam, who cannot refrain from believing the content of his hallucinatory experience, forms the belief that a glass fell on his kitchen floor and broke and endorses a correct account of why the glass broke.

Here, I am working under the assumption that Sam’s hallucinatory experience provides him with defeasible justification for believing that a glass fell on his kitchen floor and broke and that his knowledge that he has been drugged undercuts the support provided by that experience. Consequently, I take this case to match the second possible reading of Dellsén’s Con Man case. In addition, I believe, following Grimm, that one cannot attribute to Sam an understanding of why the glass that fell on his kitchen’s floor broke although Sam has a sufficient grasp of the explanatory link between that state of affairs and the account his hallucination prompts him to endorse. The reason is, as in the Brain Lesion case, that Sam is not justified in believing that anything requires explaining in that situation and, to enter into the game of understanding why such and such is the case, one must at least be justified in thinking that such and such is to be explained—i.e. that there exists a correct explanation of why such and such is the case.

If this is correct, why do cases such as The Con Man case tend to elicit the intuition that the subject understands why *p* is the case? The aspect of The Con Man that elicits this intuition is that Bernie’s grasp of the link between the account he endorses and the state of affairs he comes to believe is such that had Bernie been justified in believing that that state of affairs needed to be accounted for, he would be in a position to correctly account for it. That is, the strength of a case such as The Con Man case is to blur the line between two intuitive requirements on understanding: one concerning the grasp S must have of the explanatory link between H and *p* and the other concerning the justification S needs for believing that *p* is in need of being explained. Because Bernie satisfies so clearly the first of these requirements, one is tempted to attribute the corresponding understanding to him. But once more extreme cases such as the ones just discussed are considered, it becomes increasingly plausible that if S lacks justification for believing that *p* is the case, S cannot enter into the game of understanding why *p* although her grasp of the link between H—a correct account of why *p*—and *p* would be sufficient to occupy a winning position in that game.

### Evidence for the explanans

What of cases in which S is justified in believing that *p* but lacks justification for believing H’s content? Hills offers the following case:

*Great Leader*: Suppose that you read in your book that Napoleon was tactically astute, and so on, and on that basis conclude that he was a great leader. But now your history teacher, whom you regard as extremely trustworthy, tells you that Napoleon was not a great leader. Your teacher is not basing this judgement on other information or on a different interpretation of what it takes to be a great general: he simply irrationally dislikes Napoleon. You have no idea about any of this, but even so, you ignore your teacher and continue to maintain your conclusion. Just as in the previous example, you have the abilities required for understanding, your beliefs are correct and in short, you understand why Napoleon was great.^2^ (2016, p. 672)

Here it can be stipulated that S, the subject involved in that case, came to learn various facts about Napoleon—that he was tactically astute and so on—and that, therefore, she has justification for believing this to be true of that historical figure. S lacks, however, justification for believing the correct explanation of those facts about Napoleon (H)—that he was a great leader—for what her teacher tells her constitutes a rebutting defeater. Consequently, this case presents us with a situation where S is justified in believing that *p* but, because she possesses a undefeated defeater, lacks justification for endorsing an explanation H of *p* that is, as a matter of fact, the correct explanation of why *p*. Can S understand why *p* by maintaining her belief in H in such a situation? That is, does the teacher’s testimony undermine both the justification S has for believing that Napoleon was a great leader and her claim to understand the facts about Napoleon she came to learn?

According to Hills (2016) there is little doubt regarding the fact that S understands what she learned about Napoleon in the Great Leader case. For although S lacks justification for endorsing the content of H, she has the required cognitive control over the connexion between H and *p*. Hills’ notion of cognitive control concerns what may be called the grasping component of understanding. Recall that according to philosophers such as Kvanvig (2003), understanding involves grasping how the various elements of a body of information relate to each other. For Hills, to possess such a grasp is to be able to manipulate the relation between these elements in the actual circumstances and in relevantly similar circumstances—in the case of explanatory understanding the relation between H and *p*. As S possesses the type of control over the relation between H and *p* that Hills deems characteristic of understanding in the Great Leader case, she naturally concludes that this case shows that understanding why *p* by means of H does not requires having justification for believing H’s content.

I believe, however, that the case Hills offers shows something quite different. It shows, essentially, that contrary to what philosophers such as Kvanvig tend to assume, S can have the required grasp of the relation between H and *p* without thereby being justified in endorsing H’s content. But it does not follow from this that justification for endorsing H’s content is not required for gaining an understanding of why *p* by means of H. As already noted, if understanding why *p* by means of H involves grasping the explanatory and other coherence making relationships between H and *p,* it is at least *prima facie* plausible that if S grasps the connexion between H and *p* in the way required to understand why *p*, then S is justified in endorsing the content of H. This is because, if S grasps these relations, she grasps how well H can explain why *p* compared to other potential explanations and therefore grasps how well H’s content is supported. So it seems that, as Jägger puts it: “the degree to which S understands a subject matter is proportional to S’s awareness of the relative epistemic weight of the total available reasons relevant to propositions belonging to that subject matter” (2016, p. 180). Yet the Great Leader case puts pressure on the supposedly close connexion between the grasping component of understanding and the justification S has for endorsing H’s content. In that case, as noted by Hills, it is perfectly possible for S to have a complete grasp of the connexion between H and *p* without thereby being justified in believing H’s content. The reason is that, as Dellsén (2017, p. 245) suggests, in the Great Leader case, the defeating evidence S acquires is not explanatorily relevant. There is of course, a sense in which the teacher’s testimony is explanatorily relevant as it speaks for believing that the account S endorses is not the right way to account for what she learned about Napoleon. But there is another sense in which it is not directly explanatorily relevant: the teacher’s testimony is not itself something that the account endorsed by S is designed to explain. That Napoleon was a great leader bears no direct explanatory relation with the teacher’s testimony. Accordingly, S can fully grasp how well H is supported by the facts that that explanation is designed to explain and, at the same time, lacks justification for endorsing the content of that explanation because of what her teacher tells her. That she fully grasps the explanatory connexion between H and *p* does not guarantee that she is justified in believing H’s content.

What makes the Great Leader case interesting is thus that it shows that the grasping component of understanding is independent from the justification a subject has for endorsing the content of the account that she grasps. It is however an entirely different question as to whether this case manages to show that S does not need justification for believing H’s content to understand why *p* by means of H. With respect to this particular question, it seems to me that Pritchard is essentially correct in claiming that “it is hard to make sense of how an agent could possess understanding and yet lack good reflectively accessible grounds in support of that understanding” (2009, p. 33). Pritchard does not provide much detail about what it means for S to possess reflectively accessible grounds in support of her understanding of why *p*. But one reasonable reading of his claim is that understanding is incompatible with what Pritchard (2005) labels reflective epistemic luck. More precisely, that for S to understand why *p* by means of H, it has to be the case that *from S’s reflective position*, it is not a matter of luck that H is the right way to account for why *p*.

In the Great Leader case, such a condition is typically not satisfied, for given S’s reflective position—that is, given what S has access to without further empirical inquiry—it is a matter of luck that the explanation she endorses is the right way to account for the various facts she learnt about Napoleon. After all, if the teacher’s testimony is truly sufficient for defeating the justification S has for believing the content of that explanation, then from S’s reflective position, the fact that that explanation turns out to be the correct one is a matter of luck. But why think that, as suggested by Pritchard, understanding is not compatible with such reflective luck? Intuitively, gaining an understanding of why *p* by means of an explanation H involves using H to make an initially surprising fact intelligible. When S understands why *p* by means of H, *p* is no longer surprising from S’s reflective position, and this seems to be a crucial aspect of understanding. This in turns suggests that S cannot understand why *p* by means of H if her reflective position is such that from that position it is a matter of luck that H constitutes the correct way to account for why *p*. For if S is in such a reflective position, there is a clear sense in which, in spite of S’s supposed understanding of why *p*, there remains something inherently surprising for S concerning why *p*. Given what is reflectively accessible for S, it is a matter of luck that she is correct concerning why *p*. Let me consider the following situation inspired by a case initially offered by Khalifa (2017, p. 196) to illustrate this point:

*Lazy Fireman*: Suppose that Sam arrives at what remains of a house that was destroyed by a fire. Sam’s job is to examine the embers of the house in order to understand why the house burned down. He is familiar with this type of house and, given a preliminary inspection, it looks like the fire was due to a faulty breaker box. Nevertheless he is also aware that such houses can also catch fire because of a shorted grounding wire and it had often happened to Sam that contrary to initial appearances, voltmeter readings confirmed that the fire was due to a shorted grounding wire instead of a faulty breaker box. But Sam, who would prefer going home over preforming the tests that could rule out either of these possible explanations, simply maintains his initial conclusion that the fire was due to a faulty breaker box and goes home.

The Lazy Fireman case is structurally similar to the Great Leader case. Here Sam has inductive evidence that defeats his justification for believing that the fire was due to a faulty breaker box. It seems clear that without performing the required tests, he is not justified in endorsing the faulty breaker box explanation. But, as he is a particularly lazy fireman, he maintains his belief that the fire was due to a faulty breaker box. Now, if Sam’s belief that the fire was due to a faulty breaker box turns out to be true, should we credit Sam with an understanding of why the house burned down? It seems not. Although Sam may have full cognitive control over the explanatory connexion between the fire and the hypothesis he comes to endorse, it is simply implausible that he can understand why the house burned down by maintaining, out of sheer laziness, his conclusion. The reason for this is that without performing the required tests, it is a matter of luck, from Sam’s reflective position, that the faulty breaker box explanation is the right way to account for the fire. Prior to acquiring evidence that would defeat the defeater Sam possesses, he is not in a position to understand why the house burned down. Likewise, in the Great Leader case, the teacher’s testimony—assuming it defeats the justification S has for maintaining her conclusion—defeats the understanding she may have had of the facts she learnt about Napoleon. For once S’s teacher states that Napoleon was not a great leader and thereby suggests that another correct yet unknown explanation of the facts that S learnt about Napoleon exists, S is no longer in a reflective position that is such that, from that position, it is not a matter of luck that the explanation she endorses is the correct way to account for what she aims at explaining.

Once cases of lazy understanding such as the Lazy Fireman are considered, it thus becomes increasingly plausible that one cannot understand why *p* by means of H if, from one’s reflective position, it is a matter of luck that H is the correct way to account for why *p*. Let me stress, however, that accepting such a condition on understanding does not commit one to the claim, made by Zagzebski (2001), that when one understands why *p* by means of H one is in a position to know or to understand that one understands why *p*. Even if understanding why *p* by means of H requires being in a reflective position that is such that from that position it is not a matter of luck that H is the correct way to account for why *p*, other conditions exist on understanding. It is plausible, for instance, that understanding involves an accuracy requirement and depending on the nature of the grounds that are reflectively available to the subject when she understands why *p*, these grounds alone might not suffice to know that she understands why *p*.^3^ Hence, the considerations just put forward, while speaking for the conclusion that understanding involves an internalist component, leave the question of the transparency of understanding open.

## Arguments from deductive cogency

### Deductive cogency of belief

According to Dellsén (2018, 2021) one reason to reject the claim that understanding why *p* by means of H requires having justification for believing H’s content is that deductive cogency is not a requirement on belief. In particular, Dellsén proceeds from the following premises to the conclusion that understanding does not require believing H’s content with justification:

(P_1_) The set of propositions to which it would be acceptable to commit in understanding some phenomenon is deductively consistent and closed under deductive consequence. (Dellsén, 2021, p. 2480)

(P_2_) The set of propositions one believes or is prepared to believe need not be consistent and closed under logical consequence.

(C) The set of propositions to which it would be acceptable to commit in understanding some phenomenon need not be a set of propositions which it would be acceptable, for S, to believe.

Here, I will follow Dellsén in considering that if deductive cogency is a requirement on belief, then the set of propositions one believes or is prepared to believe should be consistent and closed under logical consequence. I will therefore treat deductive cogency as a rational requirement on a particular kind of attitude, the notion of acceptability involved in (P_1_) and (C) being understood in relation to the norms governing a particular type of attitude.

Dellsén (2021, pp. 2478–2479) takes (P_1_) to follow naturally from the requirement that to understand some phenomenon, one must grasp a coherent body of information. (P_1_), in essence, requires that one’s account of why *p* be logically coherent and that one be prepared, in understanding why *p* by means of that account, to endorse what logically follows from its content. As I take such a requirement to be plausible, I shall not discuss (P_1_) any further in the context of the present article and shall concentrate on the considerations put forward by Dellsén in favour of (P_2_).

Why think that deductive cogency is not a requirement on belief? As Dellsén (2018, pp. 3125–3128) himself notes, the claim that the set of propositions one believes or is prepared to believe should be consistent and closed under logical consequence enjoys a great deal of *prima facie* plausibility. It is intuitively correct that one cannot rationally believe a logically inconsistent set of propositions—that is, a set of propositions from which a contradiction can be deduced. It is also highly plausible that any rational system of belief should accept its own consequences and, as a result, be closed under logical consequence.^4^ Yet, such a requirement on belief seems to conflict with two independently plausible claims. The first claim concerns the conditions under which a subject’s credence for a set of propositions can be deemed rational. The second concerns the relation between the propositions a subject can rationally believe and the credence she can rationally adopt toward these propositions.

Probabilism is the view that, if rational, one’s credence toward a set of propositions can be represented as a probability distribution over that set. Dutch Book arguments and Representation Theorems provide strong support in favour of that view by showing how, if representable as a probability distribution over a set of propositions, one’s credence relates to preferences that satisfy intuitively rational constraints.^5^ In addition, rules of conditionalization offer a precise way to model how one’s credence toward a set of propositions should be updated in light of new evidence in a probabilistically coherent manner. The Lockean Thesis of rational belief, for its part, establishes a strict relation between the credence one can rationally adopt toward a proposition and rational belief.^6^ More precisely, this view identifies a threshold *t* such that .5 $\le$ *t* $<$ 1 and it is rationally acceptable for S to believe a proposition *p* whenever it is rational for S to have a credence for *p* above *t*; by Probabilism, the credence one can rationally adopt toward *p* is to be conceived as the subjective probability one can assign to *p* given one’s overall evidence.

The conjunction of Probabilism and the Lockean Thesis of rational belief raises a challenge for the idea that deductive cogency is a requirement on belief for, when conjoined, these claims deliver the result that the set of propositions one should believe need not be either logically consistent or closed under logical consequence. To illustrate this challenge, Dellsén (2021) considers Makinson’s (1965) Preface Paradox and Kyburg’s (1961) Lottery Paradox:

*Preface Paradox*: A historian has just finished writing a book on, for example, European emigration to North America. This historian is a responsible scholar, so let us suppose that she is epistemically justified in believing each one of the many claims she makes in the book. However, if the book is thick enough, it also seems that she would not be justified in believing that she hasn’t made at least one error somewhere in the book. (Dellsén, 2018, p. 3128).

The reason why the historian does not appear justified in believing that the body of her book contains no mistake is that given her overall evidence, such a claim is highly improbable. Even if the probability she can rationally assign to each claim made in her book is high, the probability of their conjunction on her overall evidence is low which, it seems, speaks for the conclusion that she lacks justification for believing their conjunction. Yet, the conjunction of those claims is a logical consequence of the body of propositions the historian is justified in believing and therefore the Preface Paradox elicits the intuition that the historian is not required to believe what logically follows from her system of beliefs.

The Lottery Paradox presents us, for its part, with a situation in which a subject appears to be justified in believing a set of logically inconsistent propositions. Suppose that a lottery is known by S to be fair and to contain 1000 tickets (this number can be set arbitrarily high). Given what S knows prior to the lottery results, each ticket has a very high probability of being a loosing ticket. Accordingly, the conjunction of Probabilism and the Lockean Thesis suggests that for each ticket *x*, S is justified in believing that *x* is a losing ticket. But as S knows and is presumably justified in believing that the lottery is fair—i.e. that one lottery ticket is a winning ticket—S is justified in believing a set of logically inconsistent propositions.

The plausibility of (P_2_) thus depends on the apparent inconsistency, illustrated by the paradoxes just considered, between the claim that deductive cogency is a requirement on belief on the one hand and Probabilism and the Lockean Thesis on the other hand, the motivation for endorsing (P_2_) stemming from the independent plausibility of Probabilism and the Lockean Thesis. Yet, as Dellsén (2021, pp. 2482–2483) remarks, the Lockean Thesis is itself a controversial claim and it is far from clear that the plausibility of this claim outweighs the *prima facie* plausibility of the claim that deductive cogency is a requirement on belief. Nevertheless Dellsén holds that:

To deny the Lockean thesis in this form is to say, first, that someone could be justified in believing a proposition that is, by her own rational lights, as improbable as you like (provided its probability is not zero); and, second, that someone could fail to be justified in believing a proposition that is, by her own rational lights, as probable as you like (provided its probability is not one). It seems to me that this would do much more violence to our pretheoretical conceptions about justification and rationality than rejecting anything about the connection between understanding and justification. (2021, p. 2483)

According to some philosophers, however, endorsing the view of rational belief Dellsén relies on would do much violence to our pretheoretical conceptions about justification and rationality for reasons that are independent of the question of whether deductive cogency is a requirement on belief. Buchak (2014) for instance argues that our intuitive judgements concerning rational belief conflict with the Lockean Thesis when the evidence possessed by a subject in favour of a proposition *p* is purely statistical with respect to that claim.^7^ Alternative accounts of rational belief such as McCain’s (2014) and Poston’s (2014), which conceive of (full) belief justification in terms of explanatory considerations instead of probabilistic considerations manage to deliver intuitive results in the type of cases discussed by Buchak and preserve the requirement of deductive cogency on belief. Accordingly, the Lockean Thesis which (P_2_) depends on cannot simply be vindicated by the type of considerations put forward by Dellsén, for there are independent reasons to believe that alternative accounts of rational belief which preserve the idea of belief’s deductive cogency are preferable to the Lockean Thesis.

Note that the rejection of the view of rational belief which (P_2_) depends on does not necessarily entail rejecting the idea that the propositions one can rationally believe are determined by the magnitude of the probabilistically coherent credence one can adopt toward those propositions. Leitgeb (2014, 2015) offers an account of rational belief—the Humean Thesis—which encapsulates this idea while remaining consistent with the claim that deductive cogency is a requirement on rational belief. According to Leitgeb’s account, the propositions that a subject can rationally believe are those propositions toward which she can adopt a stable credence above a threshold *t*, and this view is shown by Leitgeb (2015, pp. 166–173) to preserve the idea that the propositions one believes or one is prepared to believe should be logically consistent and closed under logical consequence.

For Dellsén’s (2021, p. 2483), Leitgeb’s proposal is however unconvincing when compared to the Lockean Thesis, for according to Leitgeb’s (2013) original proposal, the threshold for rational belief is set, in a particular context of reasoning, by the stably high credence a subject can rationally adopt toward the logically strongest proposition relevant in the context. Consequently, this account “makes the choice of threshold depend on factors about the agent that seem to me to be entirely irrelevant to whether she would be justified in believing the propositions in question” (Dellsén, 2021, p. 2483). Yet, in later versions of his account, Leitgeb (2014, 2015) opts for a contextualist approach according to which the stable credence that fixes the threshold for rational belief depends on the stakes involved in the context of reasoning as well as the doxastic standards that apply in that context and it is far from obvious that such factors are irrelevant to whether a subject is justified in believing a particular claim. At any rate, the mere fact that there exist plausible alternatives to the Lockean Thesis shows the inherent weakness of Dellsén’s argument in favour of (C). As outlined in the previous sections, there are independent reasons to endorse the view that understanding why *p* by means of H requires having justification for believing H’s content and if (P_1_) is correct, this might be taken as providing additional support for rejecting the Lockean Thesis of rational belief in favour of other plausible and well motivated accounts that have been defended. (P_1_)’s truth in conjunction with Probabilism simply isn’t sufficient to warrant the conclusion that understanding does not require doxastic justification and that the Lockean Thesis of rational belief is correct.

### Deductive cogency of acceptance

In Dellsén’s view (2018, 2021), (P_1_) and (P_2_) do not only support the conclusion that understanding why *p* by means of H does not require having justification for believing H’s content. Their conjunction supports conceiving of the type of propositional commitment involved in understanding in terms of acceptance instead of belief. This is because, according to what Dellsén argues, deductive cogency is a requirement on acceptance rather than belief. The notion of acceptance Dellsén relies on is borrowed from Cohen, according to whom:

To accept that *p* is to have or adopt a policy of deeming, positing, or postulating that *p* – *i.e.* of including that proposition or rule among one’s premises for deciding what to do or think in a particular context, whether or not one feels it to be true that *p*. (Cohen, 1992, p. 4)

Acceptance is thus, in Cohen’s view, an attitude that can be adopted in a given context of inquiry toward a proposition regardless of whether one believes this proposition to be true or “feels it to be true” as he puts it. In addition, the set of propositions that one accepts or is prepared to accept in a particular context of reasoning should be consistent and closed under logical consequence as opposed, according to (P_2_), to the set of propositions one believes or is prepared to believe.

Of course, if what I claimed regarding (P_2_) in the previous section is correct, then (P_1_) in conjunction with the claim that deductive cogency is a requirement on acceptance hardly provides reasons to think that the type of propositional commitment involved in understanding is best conceived of in terms of acceptance. But there are independent reasons, according to Dellsén, for thinking that acceptance of H’s content suffices for understanding. Consider the following case:

*String Theorist*: Carrie is a theoretical physicist in a nearby possible world (perhaps this one) in which string theory is true. Carrie has built her career around using string theory to explain various known phenomena about the natural world, and has become one of the world’s leading contributors in the field because of her unmatched insight into the theory and its applications. Moreover, she has adopted the policy of treating string theory as given in her scientific endeavours – using it in explanations of various natural phenomena – and thus accepts string theory for explanatory purposes. However, like many other physicists, Carrie has significant methodological reservations about string theory in its current form, and therefore is not disposed to feel that string theory is even approximately true. In other words, Carrie does not believe that string theory is even approximately true.

Here, Dellsén argues, the fact that Carrie does not believe string theory to be true or even approximatively true is no reason to think that she is not in a position to understand the natural phenomena she aims at understanding by means of that theory. The reason is that acceptance of that theory is sufficient for understanding.

Conceiving of the propositional commitment involved in understanding in terms of acceptance rather than belief, however, does not come without problems. One aspect that distinguishes acceptance from belief according to philosophers such as Bratman (1992) or Cohen (1992) is that acceptance is tied to a particular context of reasoning. This aspect is also acknowledged by Dellsén who talks about propositions a subject treats as true in a particular context of explaining. Yet, the contextual nature of acceptance raises serious issues when it comes to elucidating the propositional commitment involved in understanding. Suppose that Carrie treats string theory as true in attempting to explain some natural phenomenon and suppose, for the sake of the argument, that her acceptance of the claims constitutive of that theory suffices for her to gain an understanding of that phenomenon. How should we conceive of Carrie’s cognitive standing with respect to the explained phenomenon outside of that particular context? Does Carrie still understand it when she is not treating the claims constitutive of string theory as true to explain it? Intuitively, it seems that if a subject has understood a particular phenomenon by means of some account of it, she retains that understanding (provided that the standards of epistemic appraisal haven’t changed) although she is not in the process of explaining that phenomenon. But if the propositional commitment involved in understanding is conceived of in terms of contextual acceptance, it becomes quite hard to account for that intuition. Presumably, Carrie, in the String Theorist case, does not go on treating the propositions of string theory as true in contexts which do not pertain to the explanatory use she makes of that theory. Nevertheless she retains the understanding of natural phenomena gained thanks to that theory.

What seems to motivate Dellsén’s reading of such cases is a kind of scepticism regarding the justification one can have for believing the content of theories such as string theory. One may indeed reason as follows: such theories are best conceived with respect to their content as logically closed set of propositions and many propositions that constitute their content are simply too improbable on our overall evidence for us to be justified in believing that they are true. Yet, these theories provide us with an understanding of natural phenomena and, as a result, the type of commitment involved in the understanding such theories provide is best conceived of in terms of contextual acceptance whose conditions of justification are quite different from that of belief. Such a reasoning, however, heavily relies on the Lockean Thesis of rational belief and as outlined in the previous section that assumption is disputable. If, as some philosophers claim, it is the explanatory merits of a particular theory rather than the conditional probability of its content that determines whether one is justified in endorsing that theory, such scepticism would be misplaced.

A further issue with the use Dellsén makes of cases such as the String Theorist concerns the distinction that can be drawn, as Lawler (2021) argues, between propositions that facilitate one’s understanding of why *p* and propositions that are elements of one’s understanding of why *p*. Lawler develops that distinction in response to cases discussed by Elgin (2004, 2017) of understanding induced by idealizations such as the ideal gas law that are known to be false. Nevertheless that distinction finds application with respect to the problem just raised for Dellsén’s reading of the String Theorist case. As outlined, conceiving of the commitment involved in understanding in terms of acceptance raises certain problems due to the contextual nature of acceptance and these problems can be viewed as adding support to the distinction Lawler is drawing. Supposing that, in the String Theorist case, Carrie only contextually accepts string theory, how should the content of her understanding of the natural phenomena explained by string theory be viewed outside of the contexts in which she treats string theory as true? Obviously, the claims of string theory cannot be viewed as elements of the content of her understanding in such contexts for, in these contexts, Carrie does not treat these claims as true. Presumably, in these contexts, the content of her understanding consists of claims Carrie has come to *believe* about the phenomena she understands as a result of her use of string theory. But if this is true, then why think that the claims of string theory that are only contextually accepted by Carrie in explanatory contexts are elements of the understanding she gains at all? It seems, after all, that the claims she contextually accepts are mere facilitators of the understanding she gains while the claims she has justification to believe about the natural phenomena she attempts to explain can be adequately conceived as elements of the content of her understanding—these claims being, plausibly, less abstract claims linking Carrie’s observations with the content of string theory. Consequently, even if one insists on describing Carrie as contextually accepting the content of string theory, there is a clear sense in which the propositions she accepts, because they are only contextually accepted, cannot be regarded as elements of the content of the understanding she gains of natural phenomena. The claims she accepts are mere facilitators of her understanding while the content of that understanding is limited to claims she is justified in believing in any context.

## Conclusion

What I hope to have shown in the present paper is that in spite of the counter-examples recently presented in the literature, the requirement that to understand why *p* by means of H, a subject needs justification for believing H’s content remains a plausible one. The upshot of the considerations that have been put forward is that understanding, like knowledge, has a rational dimension which is tied to the justification the understander has for believing claims that can promote her understanding of phenomena. In fact, as noted by some philosophers, the justification requirement on understanding is even more demanding than the corresponding requirement on knowledge (at least given certain theories of knowledge), for that requirement, when it comes to understanding, is tied to the subject’s reflective position.

## Footnotes

1 In particular, understanding is held by a number of philosophers to be compatible with certain forms of knowledge-undermining luck. See for instance Kvanvig (2003) and Pritchard (2010).

2 Dellsén (2017, p. 242) offers a similar case in which the justification S has for believing H is not defeated by testimonial evidence but by inductive evidence speaking for the conclusion that S, who endorses H, is probably wrong concerning why *p*.

3 The grounds S has for endorsing a particular explanation might be conceived, for instance, as non-factive mental states.

4 As outlined by Dellsén (2018, p. 3126), the requirement of deductive cogency on belief also explains the force of deductive arguments and, in particular, the rational pull of deductions *ad absurdum*.

5 See for instance Ramsey (1926), Savage (1954) and Joyce (1999).

6 See Foley (1992, 2009) for a defense of that view.

7 See also Smith (2016, 2018).
